# Spatiotemporal Epidemiology of Oropouche Fever, Brazil, 2015–2024

**DOI:** 10.3201/eid3010.241088

**Published:** 2024-10

**Authors:** Paulo Ricardo Martins-Filho, Thialla Andrade Carvalho, Cliomar Alves dos Santos

**Affiliations:** Federal University of Sergipe, Aracaju, Brazil (P.R. Martins-Filho, T.A. Carvalho);; Government of Sergipe State, Aracaju (C.A. dos Santos)

**Keywords:** Oropouche, arboviruses, viruses, vector-borne infections, disease hotspot, Brazil

## Abstract

We assessed the spatiotemporal dynamics of Oropouche fever in Brazil during 2015–2024. We found the number of cases substantially increased during that period, particularly in the Amazon region. Our findings underscore the need for improved surveillance and public health measures in response to the disease’s potential spread beyond endemic areas.

Oropouche fever is an emerging arboviral disease caused by Oropouche virus (OROV) and primarily transmitted by *Culicoides paraensis* biting midges. OROV is endemic to the Americas, predominantly the Amazon region of Brazil; estimates show ≈5 million persons live in areas at high risk for OROV transmission (*1*). Despite potential widespread transmission, Oropouche fever has been neglected, and limited data complicate implementation of effective disease control measures. In Brazil, OROV infection has caused numerous outbreaks, particularly in the Amazon region (*2*), where the climate and forest environment lead to vector proliferation. In 2024, the Pan American Health Organization and World Health Organization issued alerts of increased cases outside the Amazon (*3*) and possible vertical transmission events (*4*). Geographic spread affecting both rural areas and densely populated urban centers in non–Amazon region states underscores the virus’ adaptability to varied environments and highlights the urgent need for intensified surveillance and proactive prevention strategies. We assessed the spatiotemporal dynamics of Oropouche fever in Brazil during January 2015–March 2024.

We used anonymized data from the General Coordination of Arbovirus Surveillance of the Ministry of Health (protocol no. 25072.020334/2024-62) and included cases confirmed by reverse transcription PCR or enzyme immunoassay. We extracted information on sex, age, symptom onset, sample collection date, diagnostic method, and location of case notification. We mapped case distributions and calculated cumulative incidence rates per 100,000 inhabitants by using 2022 population census data. We identified high-risk clusters through retrospective spatiotemporal scanning by using SaTScan version 10.1.3 (https://www.satscan.org), QGIS version 3.36.3 (https://qgis.org), and the discrete Poisson model adjusted for population size. For temporal analysis, we used sample collection dates as reference points, given their enhanced precision and reliability within our dataset. We ran Monte Carlo simulations for significance testing and applied the annual percentage change technique by using Joinpoint Regression Program version 5.0.2 (https://surveillance.cancer.gov/joinpoint) to analyze disease incidence trends. We considered p<0.05 statistically significant in all analyses.

During January 2015–March 2024, Brazil recorded 5,407 Oropouche fever cases; 52% were among male and 48% among female persons. Most (71.4%) cases occurred among persons 20–59 years of age. In total, 18/27 (66.7%) states and 278/5,570 (5%) municipalities reported cases. Among notified cases, 97.1% (5,252 cases) occurred in the Amazon region; only 2.9% (155 cases) were reported outside that area (Appendix Table 1). Within the Amazon, Amazonas (82.4 cases/100,000 inhabitants), Rondônia (69 cases/100,000 inhabitants), and Acre (42.2 cases/100,000 inhabitants) states had the highest incidence rates. Among non–Amazon region states, Piauí (0.8 cases/100,000 inhabitants) and Bahia (0.7/100,000 inhabitants) had the highest rates (Figure 1, panel A).

**Figure 1:**
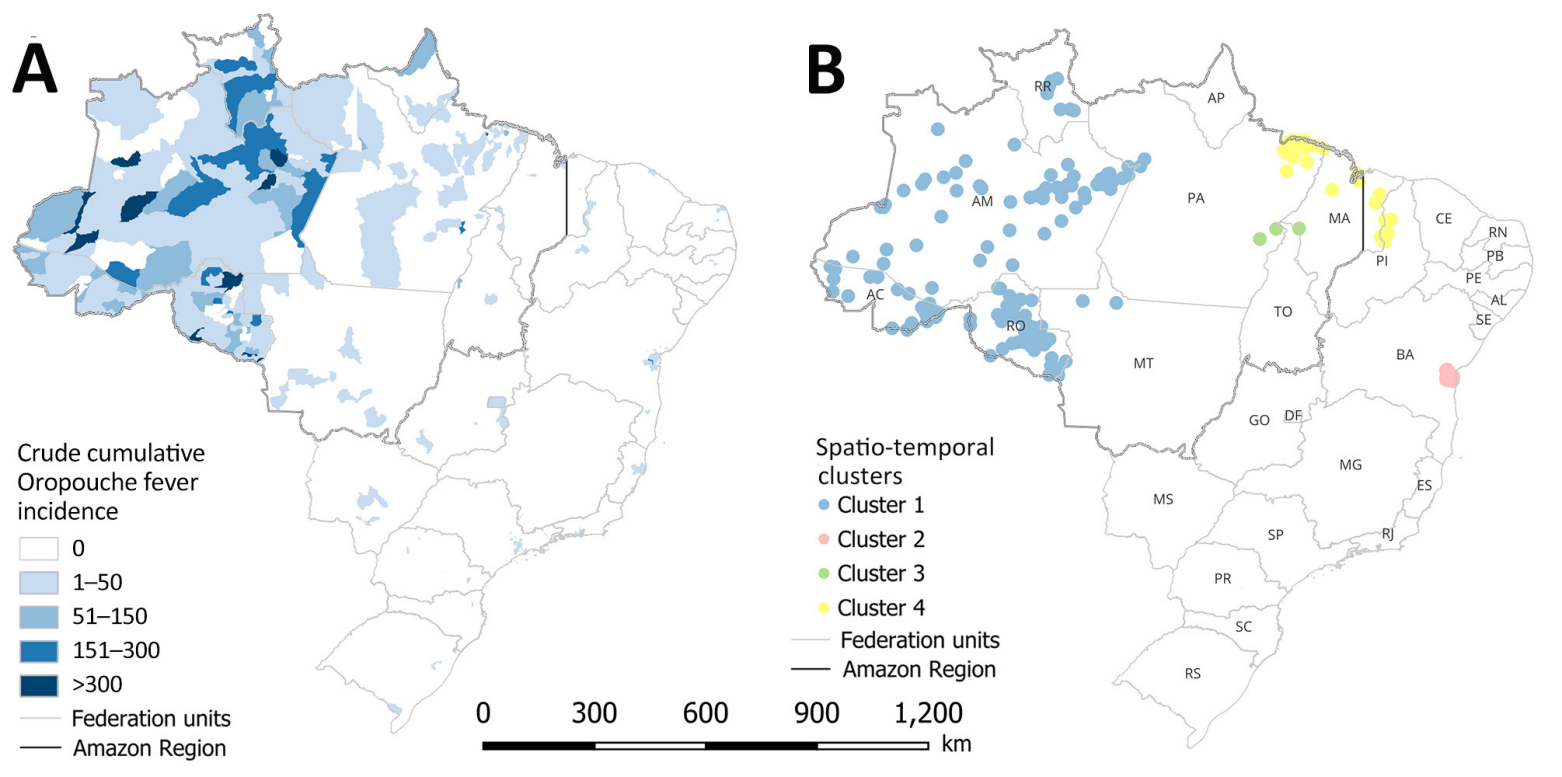
Spatiotemporal maps of epidemiology of Oropouche fever, Brazil, 2015–2024. A) Cumulative incidence (cases per 100,000 inhabitants); B) high-risk spatiotemporal clusters identified across municipalities. AC, Acre; AL, Alagoas; AM, Amazonas; AP, Amapá; BA, Bahia; CE, Ceará; DF, Federal District; ES, Espírito Santo; GO, Goiás; MA, Maranhão; MG, Minas Gerais; MS, Mato Grosso do Sul; MT, Mato Grosso; PA, Pará; PB, Paraíba; PE, Pernambuco; PI, Piauí; PR, Paraná; RJ, Rio de Janeiro; RN, Rio Grande do Norte; RO, Rondônia; RR, Roraima; RS, Rio Grande do Sui; SC, Santa Catarina; SE, Sergipe; SP, São Paulo; TO, Tocantins.

Spatiotemporal analysis identified 4 major transmission clusters: one across Amazonas, Rondônia, Acre, Roraima, and Mato Grosso starting in 2023; another in Bahia in 2024; a third in Maranhão and Pará in 2021; and a fourth in Pará, Maranhão, and Piauí in 2018 (Figure 1, panel B; Appendix Table 2). Temporal analysis also revealed a statistically significant annual increase in incidence of 145.3% (95% CI 76.5%–240.7%) and a sudden rise in reported cases during December 2023–March 2024 (Figure 2).

**Figure 2:**
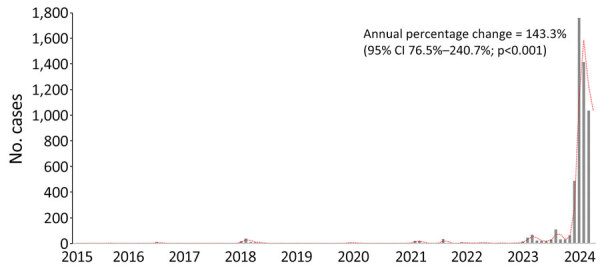
Annual cases in a study of spatiotemporal epidemiology of Oropouche fever, Brazil, 2015–2024. Bars depict distribution of cases per year and month of notification; red dotted line shows an analysis of temporal trends from January 2015 to March 2024 when case numbers rose sharply.

The first limitation of this study is incomplete travel history data, which might have missed imported cases. Another limitation is potential underdiagnosis, which might have underestimated case numbers. Finally, possible residual or cross-protection immunity could have resulted in uncertainty regarding the at-risk population.

Oropouche fever is predominantly endemic to the Amazon region, where several factors create a favorable scenario for its persistence. The humid and warm climate, complemented by dense vegetation and frequent rainfall, provide ideal conditions proliferation of *C. paraensis* midges, the primary OROV vector. Concurrently, expansion of human activities, including deforestation and urbanization, modify that vector’s natural habitats, increasing transmission risks by reducing the spaces between humans and vectors (*1*,*5*,*6*). Moreover, increasing case numbers in non–Amazon region states might be linked to heightened human mobility and climate changes that extend the geographic distribution of vector habitats. That dynamic could be exacerbated by rapid urbanization without adequate infrastructure, enabling establishment of new urban transmission hotspots (*7*,*8*). In addition, potential novel OROV reassortment could enable adaptation to new vectors or enhance virulence, further contributing to expansion to previously unaffected areas (G.C. Scachetti et al., unpub. data, https://doi.org/10.1101/2024.07.27.24310296).

Oropouche fever has symptoms similar to other arboviruses, like dengue, which contributes to underreporting and complicates accurate diagnosis (*9*). Two Oropouche fever deaths were confirmed in state of Bahia, Brazil, on July 25, 2024 (https://www.gov.br/saude/pt-br/canais-de-atendimento/sala-de-imprensa/notas-a-imprensa/2024/ministerio-da-saude-confirma-dois-obitos-por-oropouche-no-pais). Furthermore, recent reports from Pernambuco and Acre documented cases of vertical transmission, mirroring the complex epidemiologic challenges observed during the 2015–16 Zika virus outbreak (*10*). 

In conclusion, the spatiotemporal dynamics of Oropouche fever in Brazil highlight critical aspects of its epidemiology, particularly its concentration within the Amazon region and statistically significant annual incidence rate increases. Considering the geographic expansion and potential vertical OROV transmission events flagged by the Pan American Health Organization and World Health Organization, this study underscores the pressing need for an integrated surveillance and response system that includes epidemiologic surveillance and public health strategies to effectively manage the expansion of Oropouche fever in Brazil.

Appendix Additional information on spatiotemporal epidemiology of Oropouche fever, Brazil, 2015–2024.
