# Supplementary material for: Spatiotemporal Epidemiology of Oropouche Fever, Brazil, 2015–2024
Source: Emerg Infect Dis. 2024 Oct;30(10):2196–8. doi: 10.3201/eid3010.241088 (PMC11431927; doi:10.3201/eid3010.241088)
Supplement: Appendix — Additional information on spatiotemporal epidemiology of Oropouche fever, Brazil, 2015–2024. [file 24-1088-Techapp-s1.pdf]

Article DOI: https://doi.org/10.3201/eid3010.241088

EID cannot ensure accessibility for supplementary materials supplied by authors. Readers who have difficulty accessing supplementary content should contact the authors for assistance.

Spatiotemporal Epidemiology of Oropouche Fever, Brazil, 2015–2024

Appendix

Appendix Table 1. Distribution of Oropouche fever cases in Brazil from January 2015 to March 2024, categorized by sex, age group, diagnostic method, and geographic region.

Variable	No. cases (%)
Sex	
M	2,812 (52.01)
F	2,592 (47.94)
Unknown	3 (0.06)
Age group, y	
0–9	211 (3.90)
10–19	801 (14.81)
20–59	3,863 (71.44)
≥60	521 (9.64)
Unknown	11 (0.20)
Diagnostic method	
Enzyme immunoassay	433 (8.01)
Molecular biology (RT-PCR)	4,974 (91.99)
State of disease notification	
Amazonian states	
Acre (AC)	350 (6.47)
Amazonas (AM)	3,248 (60.07)
Amapá (AP)	39 (0.72)
Maranhão (MA)	5 (0.09)
Mato Grosso (MT)	1 (0.02)
Pará (PA)	335 (6.20)
Rondônia (RO)	1,091 (20.18)
Roraima (RR)	177 (3.27)
Tocantins (TO)	7 (0.13)
Non-Amazonian states	
Bahia (BA)	104 (1.92)
Espírito Santo (ES)	1 (0.02)
Goiás (GO)	5 (0.09)
Piauí (PI)	27 (0.50)
Paraná (PR)	5 (0.09)
Rio de Janeiro (RJ)	1 (0.02)
Rio Grande do Norte (RN)	5 (0.09)
Santa Catarina (SC)	3 (0.06)
São Paulo (SP)	3 (0.06)

Appendix Table 2. Brazilian municipalities included in high-risk spatiotemporal clusters for Oropouche fever from January 2015 to March 2024*

Clusters	States	No. cases	RR	p-value	Period	Municipalities
Cluster 1	Amazonas, Rondônia, Acre, Roraima and Mato Grosso	4,732	60.38	<0.001	2023 to 2024	Canutama, Lábrea, Tapauá, Humaitá, Porto Velho, Candeias do Jamari, Coari, Itapuã do Oeste, Carauari, Manicoré, Tefé, Alvarães, Alto Paraíso, Cujubim, Juruá, Rio Crespo, Codajás, Ariquemes, Boca do Acre, Buritis, Machadinho D’Oeste, Itamarati, Monte Negro, Anori, Nova Mamoré, Beruri, Cacaulândia, Campo Novo de Rondônia, Anamã, Theobroma, Novo Aripuanã, Jaru, Guajará-Mirim, Fonte Boa, Porto Acre, Acrelândia, Jutaí, Caapiranga, Ouro Preto de Oeste, Plácido Castro, Benjamin Constant, Maraã, Bujari, Rio Branco, Careiro, Teixeirópolis, Senador Guiomard, Santo Antônio do Içа, Sena Madureira, Manacapuru, Ji-Paraná, Tonantins, Manaquiri, Urupá, Capixaba, Rondolândia, Novo Airão, Borba, Presidente Médice, Iranduba, Manoel Urbano, Seringueiras, São Miguel do Guaporé, Eirunepé, Castanheiras, Ministro Andreazza, São Paulo de Olivença, Manaus, Nova Brasilândia D’Oeste, São Francisco do Guaporé, Careiro da Várzea, Envira, Novo Horizonte do Oeste, Cacoal, Barcelos, Xapuri, Rolim de Moura, Alta Floresta D’Oeste, Colniza, Nova Olinda do Norte, Costa Marques, Santa Luzia D’Oeste, Tabatinga, Espigão D’Oeste, Autazes, Pimenta Bueno, Rio Preto da Eva, Primavera de Rondônia, Feijó, Alto Alegre dos Parecis, Atalaia do Norte, Brasiléia, Epitaciolândia, Presidente Figueiredo, Tarauacá, Assis Brasil, Itacoatiara, São Gabriel da Cachoeira, Chupinguaia, Urucurituba, Silves, Ipixuna, Corumbiara, Maués, Vilhena, Cerejeiras, Apiacás, Colorado do Oeste, São Sebastião do Uatumã, Boa Vista dos Ramos, Pimenteiras do Oeste, Urucará, Cabixi, Jordão, Barreirinhas, Rodrigues Alves, Cruzeiro do Sul, Rorainópolis, Porto Walter, Mâncio Lima, Parintins, Marechal Thaumaturgo, São Sebastião da Baliza, Caroebe, Faro, Nhamundá, Caracaraí, Iracema, Juruti, Mucajaí, Oriximiná, Cantá
Cluster 2	Bahia	76	7.72	<0.001	2024	Teolândia, Wenceslau Guimarães, Presidente Tancredo Neves, Gandu, Piraí do Norte, Ituberá, Mutuípe, Igrapiúna, Laje, Taperoá
Cluster 3	Maranhão and Pará	31	37.63	<0.001	2021	Imperatriz, São Domingos do Araguaia, Curionópolis
Cluster 4	Pará, Maranhão and Piauí	64	2.62	<0.001	2018	São Luís, Rosário, Governador Newton Bello, Anapurus, Afonso Cunha, Viseu, Augusto Corrêa, Garrafão do Norte, Teresina, Altos, Capanema, Peixe-Boi, Nova Timboteira, São João de Pirabas, São Domingos do Capim, Igarapé-açu, Terra Alta, Curuça, Amarante, Benevides, Vigia, Santa Bárbara do Pará, Maracanã, Agricolândia, Palmeirais, São Francisco do Pará, Inhangapi, Castanhal, Tomé-Açu, Bujaru

*RR, relative risk
